# Role of exosomes in the development, diagnosis, prognosis and treatment of hepatocellular carcinoma

**DOI:** 10.1186/s10020-023-00731-5

**Published:** 2023-10-17

**Authors:** Meijin Liu, Zhonghong Lai, Xiaoying Yuan, Qing Jin, Haibin Shen, Dingyu Rao, Defa Huang

**Affiliations:** 1 Ganzhou Jingkai District People’s Hospital, Ganzhou, China; 2 Department of Traumatology, First Affiliated Hospital of Gannan Medical University, Ganzhou, China; 3 Laboratory Medicine, First Affiliated Hospital of Gannan Medical University, Ganzhou, China; 4 Department of Cardiothoracic Surgery, First Affiliated Hospital of Gannan Medical University, Ganzhou, China

**Keywords:** Hepatocellular carcinoma, Exosomes, Proliferation, Migration and transfer, Diagnosis, Cellular drug resistance

## Abstract

Hepatocellular carcinoma (HCC) is the most common primary liver cancer. It is characterized by occult onset resulting in most patients being diagnosed at advanced stages and with poor prognosis. Exosomes are nanoscale vesicles with a lipid bilayer envelope released by various cells under physiological and pathological conditions, which play an important role in the biological information transfer between cells. There is growing evidence that HCC cell-derived exosomes may contribute to the establishment of a favorable microenvironment that supports cancer cell proliferation, invasion, and metastasis. These exosomes not only provide a versatile platform for diagnosis but also serve as a vehicle for drug delivery. In this paper, we review the role of exosomes involved in the proliferation, migration, and metastasis of HCC and describe their application in HCC diagnosis and treatment. We also discuss the prospects of exosome application in HCC and the research challenges.

## Introduction

Liver cancer is one of the most common deadly malignancies, and patients are often diagnosed at an advanced stage, affecting treatment effectiveness. Depending on the tissue type, primary liver cancer can be divided into HCC, bile duct cell carcinoma, and mixed liver cancer, with more than 90% of all liver cancer cases being hepatocellular carcinoma. With 840,000 newly reported cases and at least 780,000 deaths each year (Couri and Pillai 2019), HCC is the fifth most common cancer in the world and the third leading cause of cancer death globally (Bray et al. 2018; Anwanwan et al. 2020). The mechanism of HCC formation is still unclear, and risk factors include chronic hepatitis B and C, alcohol addiction, metabolic liver disease (especially non-alcoholic fatty liver disease), and exposure to dietary toxins such as aflatoxin and aristolochic acid (Yang et al. 2019). The insidious onset of HCC, with more than 85% of patients detected at intermediate to advanced stages, makes treatment more difficult, and the prognosis is often unsatisfactory (Jiang et al. 2019). Currently, the screening and diagnostic methods for HCC include imaging, pathological biopsy, tumor marker testing, and combination with clinical symptoms. However, imaging depends on the level of the operator, with its sensitivity at only 60–80% and specificity at 90% (Singal et al. 2017). Although the sensitivity and specificity of pathological biopsies are considerable, this approach is often not accepted by patients and clinicians because it is invasive to patients. Alpha-fetoprotein (AFP) is currently recognized as the ideal serum protein marker for diagnosing hepatocellular carcinoma. However, some studies have shown that the sensitivity of the AFP test in hepatocellular carcinoma diagnosis is about 60%, and the specificity is only 80% (Marrero et al. 2009; Lok et al. 2010). Therefore, there is an urgent need to find a new, non-invasive, specific, and sensitive biomarker.

**Fig. 1:**
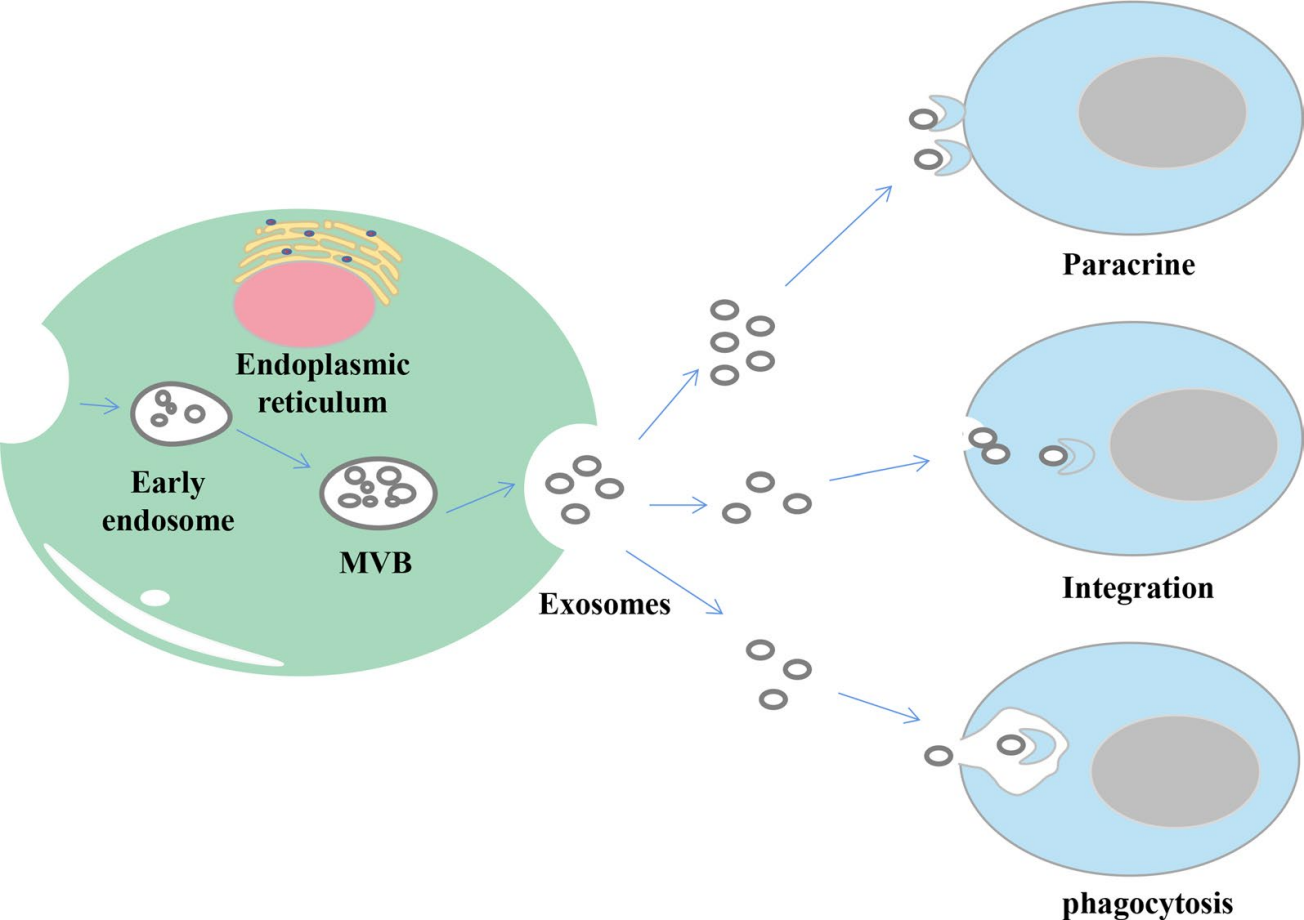
Intercellular signal exchange between exosomes and receptor cells. Exosomes begin as multivesicular vesicles (MVB), which invaginate the cytoplasmic membrane and then develop into early endosomes. The endosomes then bud inwards to form multivesicular bodies, which is to migrate to the cell surface and fuse with the plasma membrane, releasing the exosomes outside the cell in a cytosolic vomit. Exosomes release cargo molecules into recipient cells for cell-mediated delivery primarily through paracrine secretion, exosome fusion, and phagocytosis

Discovered in 1983 by Australian scientist Johnstone and colleagues, exosomes are small vesicles with a double-layer membrane structure of 4–160 nm in diameter and a density of 1.10–1.14 g/ml found in mature sheep reticulocytes cultured in vitro (Johnstone et al. 1987; Kalluri and LeBleu 2020). It is reported that exosomes are widely present in various body fluids, including but not limited to blood, urine, alveolar lavage, cerebrospinal fluid, saliva, breast milk, amniotic fluid, bile, peritoneal fluid, semen, cell culture supernatant (Zhang et al. 2015; Lasser et al. 2011; Pisitkun et al. 2004; Admyre et al. 2007; Levanen et al. 2013; Street et al. 2012; Xiao et al. 2016; Tokuhisa et al. 2015; Li et al. 2016a; Weng et al. 2016). Although the mechanism of exosome formation and secretion is unknown, exosomes carry a variety of molecules, including proteins, nucleic acids, and lipids, and are involved in various cellular biological functions. Most experimental evidence suggests that exosomes release cargo molecules into recipient cells for cell-mediated delivery primarily through paracrine secretion, exosome fusion, and phagocytosis (Alvarez-Erviti et al. 2011; Morelli et al. 2004) (Fig. 1). Exosomes not only can diffuse to the immediate cell area but also can be transported through the circulatory system to distal sites, carrying genetic information to the recipient cells, activating relevant signaling pathways, and visually reflecting the status of the recipient cells. Exosomes, a component of various body fluids, are secreted by a variety of cell types, including B lymphocytes, T cells, mast cells, dendritic cells, mesenchymal stem cells, endothelial cells, and tumor cells (Lorenc et al. 2020). Both normal and tumor cells are found to secrete exosomes, which can somewhat reflect the donor cells' physiological and pathological status and are regulated by the donor cells (Suetsugu et al. 2013; Minciacchi et al. 2015). Tumor cells secrete exosomes containing intra-tumor cell-associated molecules that alter the tumor microenvironment and receptor target cells, thereby affecting adjacent cells or specific distant cells to become cancerous (Kong et al. 2019). Tumor exosome (TEX) membranes released by tumor cells have a lipid bilayer containing by proteins, nucleic acids, cholesterol, phosphatidylserine, ceramides, and sphingolipids. (Lorenc et al. 2020). TEX plays a vital role in the proliferation, migration, and metastasis of tumor cells and is of great significance to the disease's diagnosis, treatment, and prognosis. It has also been shown that TEX is important in many tumor-related diseases. For example, the exosomal protein GPC1 was significantly higher in serum samples from pancreatic cancer patients than in normal subjects (Melo et al. 2015). Exosomes derived from hypoxic oral squamous cell carcinoma cells (OSCC) accelerate OSCC cell invasion and migration with the involvement of HIF1α and HIF2α (Li et al. 2016b). Patients with malignant melanoma had significantly higher concentrations of exosomes in their peripheral blood compared to the healthy controls, and secreted exosomal molecules were transported to the liver, explaining the occurrence of liver metastases in melanoma patients (Eldh et al. 2014).

Exosomes can also be used as a biomarker for the early diagnosis of HCC (Sasaki et al. 2019). Exosomes are detectable in a wide range of body fluids as they contain various tumor-secreted proteins in which tumor cells secrete more exosomes than normal cells into body fluids (Keerthikumar et al. 2016). In addition, tumor-derived exosomes not only reflect the molecular characteristics and phenotype of tumor origin but also the molecules transferred from exosomes are protected from degradation by a double layer of lipids to reflect the disease progression in real-time (Vanni et al. 2017). Detection of specific proteins in exosomes is highly sensitive and non-invasive compared to other methods (Keerthikumar et al. 2016; Shwetha and Smitha 2019). Many studies are focusing on the diagnostic role of exosomes in hepatocytes. However, the mechanism and role of exosomes in the development of HCC is less studied. This review summarizes the role of exosomes in the proliferation, migration, and metastasis of HCC, which extends to their diagnostic, therapeutic, and prognostic roles. Finally, we also discuss the prospects of exosome application in hepatocellular carcinoma and the research difficulties.

## Exosome cargo content and HCC

Exosomes contain a variety of cellular components, including a range of proteins, mRNAs, miRNAs, lncRNAs and DNA molecular cargoes (Zhang et al. 2018). There are several reports that exosomes include many proteins. Wang et al. used protein analysis to identify 129 proteins present in exosomes of HCC origin (Wang et al. 2017). Among these proteins, a large number of adenylate cyclase-associated protein 1 (CAP1) is contained in exosomes of HCC cells with high potential for metastasis. They suggested that CAP1 is associated with metastasis and recurrence of HCC. He et al. reported that 213 proteins were detected in exosomes derived from HCC cell lines (He et al. 2015). Interestingly, highly malignant HCC cell-derived exosomes contain high levels of MET proto-oncogenes, receptor tyrosine kinases (MET), S100 calc-binding protein A4 (S100A4), S100A10, S100A11, CAVin 1 (CAV1), and CAV2. They found that MET signaling plays a role in controlling cell migration and invasion, while CAV1 and CAV2 increase cell migration. Ye et al. found that High mobility group box 1 (HMGB1) is expressed on the membrane of HCC-derived exosomes and binds to toll-like receptor-2 (TLR-2), TLR-4, TLR-9 and advanced glycation end products (RAGE) with high affinity, leading to tumor cell survival, expansion and metastasis (Ye et al. 2018). This exosomal HMGB1-TLR-2/4-MAPK pathway may contribute to the prevention and treatment of immune tolerance in HCC.

Sohn et al. found that exosomes miR-18a, miR-221, miR-222 and miR-224 were significantly higher in serum from hepatocellular carcinoma sources than from cirrhotic patients (Sohn et al. 2015). Previous reports have shown that miR-18a induces the proliferation and development of HCC in women by decreasing the level of estrogen receptor α (Liu et al. 2009). miR-221 promotes hepatocarcinogenesis by inducing dysregulation of transcript 4 (DDIT4) through DNA damage (Pineau et al. 2010). miR-222 is associated with the migration of HCC cells through activation of the AKT signaling pathway (Wong et al. 2010). Wang et al. showed that stellate cell-derived exosomes could provide miR-335-5p cargo to recipient HCC cells, inhibit HCC cell proliferation and invasion in vitro, and induce HCC tumor shrinkage in vivo (Wang et al. 2018a). Takahashi et al. found that HCC-derived exosomes were enriched for lncRNA-ROR (Takahashi et al. 2014). Previous reports have shown that lncRNA-ROR expression is elevated in pluripotent stem cells and plays a role in the derivation of pluripotent stem cells (Loewer et al. 2010). Li et al. showed that lncRNA-FAL1 was upregulated in HCC tissues and HCC-derived exosomes (Li et al. 2018a). lncRNA-FAL1 accelerated HCC cell proliferation and migration by competitively binding to miR-1236 as a competing endogenous RNA (ceRNA) mechanism.

## The role of exosomes in HCC proliferation

The tumor microenvironment consists of components such as endothelial cells, immune cells, mesenchymal stromal cells (MSCs), fibroblasts, extracellular matrix, and soluble factors, all essential in supporting and regulating tumor growth (Hanahan and Weinberg 2011; Quail and Joyce 2013). Many studies have shown that exosomes isolated and purified from various body fluids can transmit biological information between tumor-to-tumor and tumor-to-tumor microenvironment to influence HCC proliferation and regulate HCC development. Crosstalk and information communication between cells by exosomes promote better cell survival and growth and mediate pathological changes (Villarroya-Beltri et al. 2014).

**Fig. 2:**
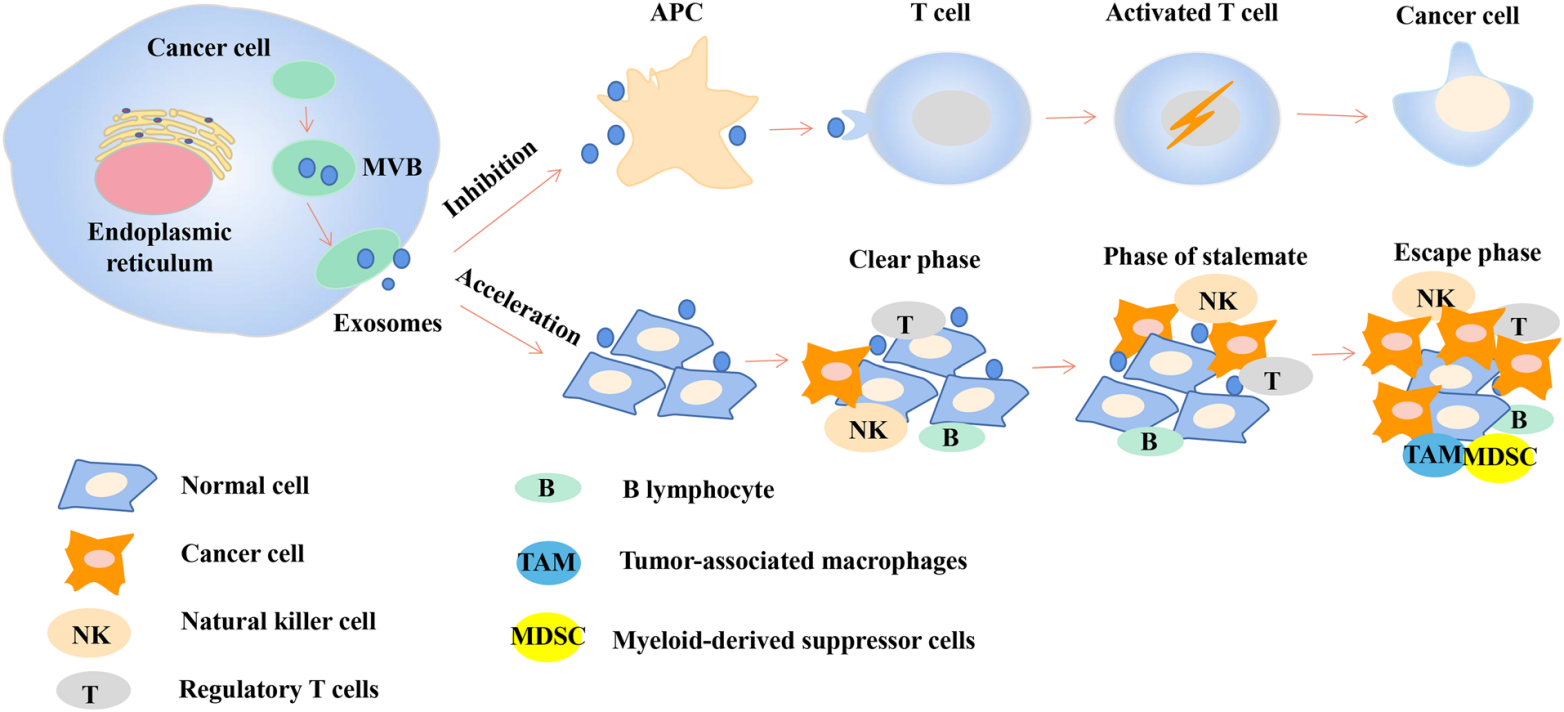
Exosomes play a dual role in HCC development. Tumor growth inhibitory effect: HCC exosomes carry tumor antigens that are taken up by dendritic cells (DCs), which activate the immune system, induce T-cell and dendritic cell-mediated anti-tumor responses, and promote anti-tumor immune responses, thereby inhibiting tumor survival and growth. Tumor growth acceleration: HCC cells promote tumor progression by facilitating immune escape of tumor cells through exosome-mediated clearance phase, staging phase, and escape phase

Exosomes play a dual role in HCC invasion and metastasis (Fig. 2). On the one hand, exosomes possess the ability to inhibit the progression of hepatocellular carcinoma. The tumor antigens carried by hepatocellular carcinoma exosomes are taken up by dendritic cells (DCs) to activate the immune system and promote anti-tumor immune response, and HCC cell-derived exosomes induce T-cell and dendritic cell-mediated anti-tumor responses, thereby inhibiting tumor survival and growth (Rafi and Omidi 2015; Rao et al. 2016). It has been shown that miR-195-containing exosomes are very effective in inhibiting the growth of hepatic cholangiocarcinoma and improving the prognosis of patients with hepatic cholangiocarcinoma (Li et al. 2017). On the other hand, exosomes can promote the growth and metastasis of hepatocellular carcinoma by inhibiting the anti-tumor immune response in multiple ways and allowing immune escape of tumor cells. Li et al. found that exosomal delivery of lncRNA FAL1 to Huh7 and HepG2 cells significantly enhanced the proliferation, migration, and invasion of HCC cells (Li et al. 2018a). Ye et al. reported that the high mobility group protein B (Breg)1 carried by hepatocellular carcinoma-derived exosomes promoted the expansion of T cell Ig and mucin domain-1 positive (TIM-1^+^) B cells, induced the secretion of TNF-α and IFN-γ factors, further inhibited the proliferation of CD8^+^ T cells, promoted the expansion of regulatory T cells, and exerted immunosuppressive effects (Ye et al. 2018). DC and tumor-derived exosomes also express a large number of major histocompatibility complex class I molecules (MHCI) and tumor markers, such as heat shock proteins (HSP), which are involved in antigen presentation and stimulation of T cells and have been shown to trigger CD8 + T cell-dependent anti-tumor responses in vitro and in vivo (Li et al. 2006). HCC exosomes can induce immunosuppression in the tumor microenvironment, assist tumor cells in undergoing immune escape, deliver pathological genetic material to target cells to influence their growth and metastasis, and even reach distant tissues and organs through blood or body fluids to build a pre-metastatic microenvironment conducive to tumor cell metastasis. In addition, tumor cells can induce immune cell death through the FasL or PD-L1/PD-1 pathway, decreasing the number of T cells and NK cells (Raposo et al. 1996). Tumor exosomes also induce suppressive immune microenvironments such as recruitment of Treg, MDSC, and these suppressive cells lead to tumor immune escape by negatively regulating CD8^+^ T cell function (Villalba et al. 2013; Olson et al. 2018). Similarly, tumor exosomes can replace tumor cells to be attacked by the immune system, thus assisting tumor cells in escaping recognition by the immune system (Gobbo et al. 2016; Chalmin et al. 2010). Exosomes can promote tumorigenesis and proliferation, in which several studies have shown that exosomes from different tumor sources have a proliferative effect on tumor cells. Golgi membrane protein 1 carried by exosomes can help promote hepatocellular carcinoma development by activating the GSK-3β/MMPs signaling pathway and inducing the proliferation of target cells (Gai et al. 2019).

## The role of exosomes in HCC migration and metastasis

Intrahepatic and distant migration and metastasis in HCC patients are key factors in poor HCC prognosis, and exosomes play an important role in HCC migration and metastasis. Exosomes, as important mediators of information transfer between hepatocellular carcinoma cells, affect not only the cells that produce them but also distant cells and their involvement in the initiation of metastasis and the formation of a pre-metastatic tumor microenvironment (Zomer et al. 2015; Aga et al. 2014), The formation of the pre-metastatic microenvironment is a prerequisite for tumor metastasis. Tumor cells can open the way for metastasis by releasing exosomes before metastasis, and when tumor cells reach the metastatic site, they can still use the exosomes to expand their territory further, and their unique intercellular communication mechanism can provide an ideal microenvironment for migration and metastasis of liver cancer. Supporting evidence suggests that exosomes are the initiators of the remodeling of the pre-metastatic microenvironment in many tumors and that exosomes can promote tumor metastasis by creating a microenvironment suitable for tumor metastasis. It has been shown that abnormal liver fibrosis enhances the recruitment of bone marrow-derived macrophages, thereby remodeling the pre-metastatic microenvironment (Costa-Silva et al. 2015). Recent studies have found that exosomes derived from highly metastatic HCC contribute to the activation of fibroblasts and promote pulmonary metastasis of HCC (Fang et al. 2018). Hepatocellular carcinoma-derived exosomes can be taken up by paraneoplastic adipocytes to activate five phosphorylated kinases, including AKT, STAT5α, GSK3 α/β, p38α, and ERK1/2 of the NF-κB signaling pathway to promote hepatocellular carcinoma invasion (Wang et al. 2018b). Tumor cells not only open the way for HCC metastasis by releasing exosomes before its arrival but also use exosomes to expand their territory further even after the tumor cells reach the metastatic site. Li et al. found that CXCR4 receptors secreted by circulating tumor cells of HCC high metastatic potential cell lines mediated the secretion of MMP-2/9 in the extracellular matrix, promoting invasion and migration of neighboring or distant low metastatic potential cell lines (Li et al. 2018b). Silva et al. demonstrated that exocytosis in pancreatic ductal adenocarcinoma (PDAC) cells containing MIF (macrophage movement inhibitory factor) induces TGF-P production by liver macrophages, which in turn upregulates hepatic stellate cell fibronectin (FN) expression while recruiting more bone marrow-derived cells and eventually forming pre-metastatic foci in liver sites (Costa-Silva et al. 2015).

Exosomes can promote tumor metastasis by enhancing tumor cells' migration and invasive ability, establishing pre-metastatic foci, and remodeling the composition of the extracellular matrix. For example, in nasopharyngeal carcinoma, exosomes from EBV-positive tumor cells are rich in Hypoxia-inducible factor-1α (HIF-1α), which effectively enhances the migration and invasion of EBV-negative tumor cells (Aga et al. 2014). The formation of pre-metastatic foci is a prerequisite for tumor metastasis, and some researchers have demonstrated that when breast cancer cells take up stromal cell-derived exosomes, they can affect the Wnt11 signaling pathway associated with tumor invasion and metastasis and influence the metastatic process (Luga et al. 2012). In addition, it has been found that angiogenesis is crucial in the development of tumorigenesis. Proteomic analysis of exosomes from malignant mesothelioma showed that exosomes are rich in pro-angiogenic factors, and when endothelial cells take up exosomes, they cause upregulation of intracellular angiogenesis-related genes, which in turn enhance the proliferation, migration, and differentiation of endothelial cells (Nazarenko et al. 2010; Park et al. 2010).

Interestingly, the role of exosomes in HCC proliferation, migration, and metastasis are strongly associated with diagnosis and prognosis. Lu et al. studied eponymous HCC-derived exosomes to trigger HCC progression and recurrence through epithelial-mesenchymal transition via the MAPK/ERK signaling pathway, which emphasized the role of exosomes in HCC metastasis and recurrence, suggesting that they are promising therapeutic and prognostic targets for HCC patients (Chen et al. 2018). Sun et al. found that exosome S100A4 derived from highly metastatic hepatocellular carcinoma cells promotes metastasis through activation of STAT3, and HCC patients with high plasma levels of exosome S100A4 also had a poorer prognosis, suggesting that exosome S100A4 is a new prognostic marker and therapeutic target for HCC metastasis (Sun et al. 2021). Furthermore, it was found that hepatocellular carcinoma-derived exosomes promote tumor progression by activating the PDK1 / AKT signaling pathway via miRNA-21 to promote hepatic stellate cell proliferation, migration and metastasis. And clinical data suggest that high levels of serum exosomal miRNA-21 are associated with longer survival time and higher vascular density in HCC patients (Zhou et al. 2018).

## Exosomes in the diagnosis of HCC

Since hepatocellular carcinoma often has no specific manifestations in the early stages, patients often miss the optimal treatment period. Tumor markers (e.g., alpha-fetoprotein), imaging, and histopathology biopsies are commonly used to diagnose hepatocellular carcinoma in clinical studies. Patients with hepatocellular carcinoma are AFP-negative up to 50%, thus AFP has low sensitivity and specificity for hepatocellular carcinoma screening (Shu et al. 2017). Imaging is highly specific; however, it has relatively low sensitivity and cannot distinguish very small tumors (Schraml et al. 2015). Histopathologic biopsies are limited by their invasive nature and high false-negative rate (Forner et al. 2018). Exosomes can be isolated from different body fluids (e.g., blood, urine, and ascites) in direct response to patient pathology. Exosomes from different tumor sources contain different levels of molecules, and exosomes differ from those in the original cytoplasm and can be several times higher/lower. This difference is more pronounced when compared to healthy individuals. The environment inside exosomes is relatively simple and stable compared to the complex environment in tissues and cells. Exosomes can carry various proteases or other enzymes through the bloodstream to their target targets, and thus can be used for cancer diagnosis and prognosis (Li et al. 2019).

In recent years, researchers have found that although most marker molecules, such as CD9 and CD81, are expressed in the same way, there are differences in exosomal markers from different sources. For example, exosomes from prostate and breast cancer sources express more CD63 molecules, which suggests that exosomes can be used as diagnostic markers for tumors (Yoshioka et al. 2013). An increasing number of studies have found that exosomes can be used as a marker for diagnosing various cancers and suggest that exosomes are a very promising method for detecting disease as a tumor marker. Due to the protective effect of the lipid bilayer membrane, proteins, nucleic acids, lipids, and other small molecule metabolites in exosomes are not easily degraded, so fresh exosome samples can be analyzed, and long-term preserved exosome samples can better reflect tumor information (Weber et al. 2010). Studies have also shown that the human plasma exosome glypican-1 protein can be a candidate marker for the early diagnosis of pancreatic cancer (Melo et al. 2015). Meanwhile, human plasma exosome Syndecan-1 is a suitable candidate marker for glioma detection (Indira Chandran et al. 2019), exosomal developmental regulatory gene protein (Del-1) to detect early breast cancer (Alix-Panabieres and Pantel 2016), and human plasma exosomal VNN1, CRP, FIBG, IGHA1, and AA1AG1 proteins as candidate markers for the detection of cholangiocarcinoma (Arbelaiz et al. 2017). A growing body of evidence supports the use of exosomes as a biomarker for the early diagnosis of HCC. Exosomal liquid biopsies have many advantages, such as being noninvasive, detectable in a variety of body fluids, fairly complete in response to tumor characteristics, relatively stable and easy to detect. The use of exosomal liquid biopsy is known to be effective in reducing and avoiding invasive injuries associated with puncture biopsy and tissue biopsy, while the use of exosome biopsy analysis can enrich patient choices and help monitor disease progression and treatment response. Sohn et al. found that the expression levels of miR-18a, miR-221, miR-222, and miR-224 in exosomes of Hepatitis B virus (HBV)-associated HCC patients were significantly lower than those of patients with chronic hepatitis B infection, suggesting that the above four miRNAs may be novel candidate plasma markers for detecting liver cancer (Sun et al. 2018). In addition, miR-30d, miR-140, and miR-29b in exosomes were significantly associated with the overall survival of HCC patients, suggesting that exosomal microRNAs (miRNAs) may also serve as prognostic biomarkers for HCC and guide the treatment of advanced hepatocellular carcinoma (Melo et al. 2014). In addition to miRNA, Li et al. reported that long non-coding RNA (lncRNA) could also be detected in plasma, and one of the possible mechanisms for its stable presence in blood is the protective effect of exosomes (Li et al. 2015a). Li et al. recently reported that high levels of serum exosome circRNA could distinguish HCC patients from healthy individuals (Li et al. 2015b). The above evidence suggests that exosomes may become a novel marker for HCC diagnosis.

## Exosomes in the treatment of HCC

Current drug delivery methods include macromolecular, particulate and magnetic agent carrier systems and gene carriers. However, issues of non-specific cytotoxicity, biocompatibility and delivery efficiency in individually targeted carrier systems have prompted the development of alternative methods of targeted drug delivery (Sacks et al. 2018; Thomas et al. 2003). One possible option is to select a range of nanocarriers from the patient's own body. Exosomes have been increasingly studied in this area in recent years. Exosomes have the following advantages as drug delivery systems. Derived from autologous cells, it is non-toxic and has low immunogenicity; Long circulating half-life, good permeability; particle size advantage, it can cross biological barriers (e.g., the blood–brain barrier) (Chen et al. 2016).

The application of exosomes is not only limited to the diagnosis of hepatocellular carcinoma; as natural nanoparticles with excellent biocompatibility, they are expected to be an important weapon to overcome the problems associated with hepatocellular carcinoma treatment. It has been claimed that exosomes have an ideal natural structure to selectively assemble and separate their contents, and their inherent biological functions make them suitable drug delivery vehicles (Li et al. 2016c). Chemotherapy is currently one of the important means of hepatocellular carcinoma treatment, which can control the progression of hepatocellular carcinoma to a certain extent. However, most hepatocellular carcinomas can develop some drug resistance after repeated drug treatment. Tumor drug resistance has been a barrier to cancer treatment in clinical care, and therefore exosomes have attracted great interest from researchers in drug resistance studies. Drug-resistant cancer cells may become resistant to hitherto sensitive cancer cells by releasing exosomes; this effect is attributed to the intercellular transfer of specific proteins, miRNAs, and long non-coding RNAs (Chen et al. 2014, 2015; Wei et al. 2014; Qu et al. 2016). Au et al. showed that miRNA-21-containing exosomes of fibroblast (CAF) origin confer paclitaxel resistance to ovarian cancer cells by targeting APAF1 in ovarian cancer (Au Yeung et al. 2016).

Qu et al. reported that exosomal-delivered LncRNA promotes AXL receptor tyrosine kinase (AXL) and cellular-mesenchymal epithelial transition factor (c-MET) expression by acting as a competitive endogenous RNA for miRNA-34 and miRNA-449, thereby promoting sunitinib resistance in renal cancer (Qu et al. 2016). Interestingly, exosomes may occupy the sites where antibodies bind to cancer cells through membrane binding, thus reducing the therapeutic effect of antibodies on cancer patients. For example, exosomes of high HER2 expression of breast cancer cell origin are reported to interfere with the activity of the monoclonal antibody trastuzumab in vitro, thereby affecting the therapeutic effect (Ciravolo et al. 2012). In summary, exosomes derived from cancer cells and stromal cells can promote the development of chemotherapy resistance in tumor cells.

On the contrary, it has also been found that exosomes, as natural carriers of signaling, can effectively prevent cellular drug tolerance by using exosomes to wrap anti-cancer drugs, which can improve drug efficacy and kill tumor cells (Ma et al. 2016). For example, Kim et al. found that trans-exosome-mediated paclitaxel treatment showed great potential for treating tumors (Kim et al. 2016). At the same time, Alcarez et al. used exosomes to artificially introduce siRNAs for systemic administration through targeted delivery of siRNAs for gene therapy purposes (Alvarez-Erviti et al. 2011). Tian et al. also demonstrated that artificially modified exosomes managed the delivery of adriamycin drugs to tumor cells through targeted modifications (Tian et al. 2014). The use of exosomes as therapeutic agents in clinical settings, in addition to wrapping anti-cancer drugs, and the complete depletion of circulating exosomes may potentially provide significant benefits to cancer patients. For example, Ciravolo et al. demonstrated that purified exosomes derived from cancer cells of breast cancer patients overexpressing HER2 were able to inhibit the anti-proliferative activity of trastuzumab (Ciravolo et al. 2012). In patients with advanced breast cancer, removing such exosomes significantly improved the anti-proliferative activity of trastuzumab (Marleau et al. 2012). In addition, a new light-guided targeting technology has also been developed, which enables the delivery of exosome-mediated soluble proteins to tumor cells under light guidance, and this light-guided targeting therapy makes tumor treatment more precise and safe. (Yim et al. 2016). Some researchers have reported the use of exosomes for cholangiocarcinoma treatment by encapsulating specific miRNAs (Gradilone 2017). The study showed that encapsulating specific miRNAs could also be used to treat hepatocellular carcinoma. Keisaku et al. injected exosomes isolated from human mesenchymal stem cells directly into mouse liver and found that they were able to inhibit the expression of collagen and TGF-β1 in vivo, thereby reducing carbon tetrachloride-induced liver fibrosis, and found that exosomes and their components were involved in the regeneration and migration of hepatocytes (Sato et al. 2016). Exosomes derived from MSC cells have attracted the interest of researchers in MSC-based cell therapy, and the approach is under active exploration. For example, Katakowski et al. demonstrated that miRNA-146b carried by MSCs exosomes significantly reduced the growth of glioma xenografts in a primary brain tumor model in rats (Katakowski et al. 2013). Similarly, a study reported by Ono et al. suggests that exosomal transfer miRNAs from MSCs may promote breast cancer dormancy at metastases (Ono et al. 2014).

The current literature shows no clinical trial studies using exosomes in the treatment of hepatocellular carcinoma. Some clinical studies of exosomes for the treatment of cancers other than HCC were conducted around 2000. For example, in a phase I clinical trial, Escudier et al. injected exosomes derived from autologous DCs intradermally and subcutaneously into patients with metastatic melanoma. Although the efficacy was not satisfactory, no significant toxic side effects were observed, suggesting that exosomes are safe and feasible for the treatment of metastatic melanoma (Escudier et al. 2005). In another phase I clinical trial, Morse et al. successfully activated the immune response in patients with non-small cell lung cancer (NSCLC) using DC-generated exosomes, which slowed down the progression of NSCLC to a certain extent, with 12 of the patients even seeing a halt in tumor progression for more than 232 months (Besse et al. 2016). There are still many difficulties that must be overcome in using exosomes as carriers in clinical cancer therapy, including liver cancer. First, homogeneity of exosomes is difficult to ensure. Secondly, exosomes are mainly administered by subcutaneous injection. While this method is simple and easy to use, the absorption efficiency is not ideal. However, with further research, we believe that exosomes will be widely used in the clinical treatment of HCC.

## Mechanisms of exosome drug resistance in HCC

Tumor-derived exosomes transfer multiple drug resistance-associated proteins and miRNAs (Wei et al. 2014; Corcoran et al. 2012). For example, drug-resistant cells can transmit the resistance-related molecules Ephrin type-A receptor 2 (EphA2) and transient receptor potential channel 5 (TrpC5) to non-resistant cells via exosomes, mediating the enhancement of drug resistance (Fan et al. 2018; Ma et al. 2014). Drug-resistant miRNAs include miR-100, miR-222, mir-30a, miR-24, miR-26a, and miR-27a (Safaei et al. 2005). Conversely, tumor cells can use exosomes to exocytose chemotherapeutic drugs and counteract targeted drug effects. One study confirmed that in drug-resistant human melanoma and ovarian cancer cells, intracellular drug concentrations decreased significantly with exosome release (Yang and Robbins 2011). Meanwhile, drug-resistant tumor cell-derived exosomes can deliver specific molecules to drug-sensitive tumor cells to enhance their drug resistance, as reported that after the delivery of exosomes containing sunitinib-resistant lncARSR to sunitinib-sensitive renal cancer cells, the sensitive renal cancer cells showed sunitinib resistance (Qu et al. 2016). In addition, exosomes carry components that may interfere with the binding of antibody drugs to tumors and reduce the effectiveness of tumor treatment. For example, CD20 molecules carried in lymphoma exosomes are able to bind to anti-CD20 antibodies, rendering tumor cells immune to drug attack (Aung et al. 2011). Interestingly, exosomes from stromal cells can mediate tumor drug resistance in addition to tumor cells. Yu et al. found that bone marrow mesenchymal hepatocyte-derived exosomes could render multiple myeloma cells resistant to bortezomib through a survival-related signaling pathway (Battke et al. 2011). Researchers found that drug resistance in adult neuroblastoma could be enhanced by upregulating miR-155 levels in monocytes (Challagundla et al. 2015).

In response to the exosome-mediated drug resistance mechanism, there are two approaches to ameliorate exosome resistance effectively: 1) inhibit the production and secretion of exosomes, or 2) inhibit the drug resistance molecules carried by exosomes. Because the biological properties of exosomes and the substances they carry are not well characterized and elucidated, it may take some time before specific drugs targeting exosomes become available. Drugs targeting cell surface receptors are reportedly neutralized by exosomes carrying the receptors, such as resistance to CD20 monoclonal antibodies. However, investigators have also found that if pretreated with indomethacin, they are able to restore the therapeutic effect of this immunotherapy by inhibiting the secretion of exosomes (Aung et al. 2011). Hu et al. showed that inhibiting fibroblast exosome production reversed chemoresistance in exosome-giving cancer stem cells (CSCs) (Hu et al. 2015). In addition, the investigators concluded that the drug-resistant and metastatic phenotypes could be interconverted (Kerbel et al. 1994). Therefore, exosome-mediated drug resistance amplification can directly affect metastatic foci formation in tumor cells (Zomer et al. 2015). With the use of exosome blockade in cancer, more drugs that inhibit exosome production are being screened and can be developed to overcome the drug resistance of tumor cells.

## Conclusion

HCC, as the main type of primary liver cancer, is characterized by high malignancy, strong invasiveness, multi-focus, uncomplicated metastasis, and poor prognosis. The onset of hepatocellular carcinoma is occult, whereby most patients are diagnosed with advanced liver cancer with poor prognosis and limited treatment effectiveness. In recent years, exosomes have received much attention due to their pathophysiological role in tumor progression. However, the mechanism of action and clinical application of exosomes in HCC is still poorly understood. Tumor invasion and metastasis, immune evasion, and drug resistance are the main obstacles to treating advanced HCC. Exosomes act as a bridge of cellular communication in the tumor microenvironment, leading to tumor initiation, invasion, metastasis, and drug resistance. And so, it is crucial to understand the involvement of exosomes in HCC development, including initiation, progression, immune escape, treatment resistance, and relapse after a period of remission. In the future, it is important to explore the subtle interactions between exosomes and the HCC tumor microenvironment, to study the possibility of cargo alteration in exosomes for targeted therapies, and to investigate exosome-based immunotherapeutic approaches for the mechanism of HCC development, diagnosis, and treatment. Therefore, this study is expected to provide a platform and guidance for developing new diagnostic and predictive tools as well as effective therapies for HCC patients.

Although considerable progress has been made in understanding exosomes and their cargoes, several challenges and questions remain. First, there are no standard methods for exosome isolation and identification, difficulties in exosome characterization and cargo analysis, and no ideal high-purity and efficient exosome isolation strategy, which will lead to less reproducible and convincing results. Second, raised questions include how and when exosomes arrive at pre-metastatic ecological sites during disease development and how cancer cells can release specific exosomes to maintain the plasticity and metastasis of HCC. Third, determining which exosome sources are safe and biocompatible for drug delivery systems in therapy is still a vague issue, and the drug delivery modalities and targeted modification techniques of exosomes need to be further refined in clinical applications. Finally, how can exosomes be used to prevent off-targeting during targeted therapy. In conclusion, exosome application is an attractive area of research that still needs to be explored in terms of the development, diagnosis, and HCC treatment effectiveness. However, some hurdles still exist to overcome before exosomes are ready for clinical use.

## Data Availability

Not applicable.
